# 中国肺癌低剂量螺旋CT筛查指南（2018年版）

**DOI:** 10.3779/j.issn.1009-3419.2018.02.01

**Published:** 2018-02-20

**Authors:** 清华 周, 亚光 范, 颖 王, 友林 乔, 贵齐 王, 云超 黄, 新允 王, 宁 吴, 国桢 张, 向鹏 郑, 宏 步, 印 李, 森 韦, 良安 陈, 成平 胡, 远凯 石, 燕 孙

**Affiliations:** 1 610041 成都, 四川大学华西医院肺癌中心/肺癌研究所 Lung Cancer Center/Lung Cancer Institute, West China University, Sichuan University, Chengdu 610041, China; 2 300052 天津, 天津医科大学总医院肺癌研究所 Tianjin Lung Cancer Institute, Tianjin Medical University General Hospital, Tianjin 300052, China; 3 610041 成都, 中国肺癌早诊早治专家组 China National Expert Group of Early Diagnosis and Treatment of Lung Cancer, Chengdu 610041, China; 4 100021 北京, 中国医学科学院肿瘤医院/国家癌症中心 Cancer Hospital, Chinese Academy of Medical Sciences/China National Cancer Center, Beijing 100021, China; 5 650105 昆明, 云南省肿瘤医院 Cancer Hospital of Yunnan Province, Kunming 650105, China; 6 200040 上海, 上海华东医院 Shanghai Huadong Hospital, Shanghai 200040, China; 7 610041 成都, 四川大学华西医院病理科 Department of Pathology, West China Hospital, Sichuan University, Chengdu 610041, China; 8 450008 郑州, 河南省肿瘤医院 Cancer Hospital of Henan Province, Zhengzhou 450008, China; 9 100853 北京, 中国人民解放军总医院 General Hospital of People's Liberation Army, Beijing 100853, China; 10 410008 长沙, 中南大学湘雅医院 10. Xiangya Hospital, Central South University, Changsa 410008, China

**Keywords:** 肺肿瘤, 指南, 筛查, LDCT, 高危人群, Lung neoplasms, Guideline, Screening, LDCT, High risk population

## Abstract

**背景与目的:**

肺癌是导致中国癌症死亡的首要原因。已有的研究证明低剂量螺旋CT在肺癌高危人群进行肺癌筛查能降低20%的肺癌死亡。本研究的目的是建立适合中国国情的肺癌筛查指南。

**方法:**

由国家卫计委任命的中国肺癌早诊早治专家组专家及部分非专家组专家，包括：4名胸外科专家、4名胸部影像学专家、2名肿瘤学专家、2名肺内科专家、2名病理学专家和2名流行病学专家，共同参与了本指南的制定工作。专家们在系统评价了美国NLST和中国农村肺癌LDCT筛查结果及经验，并达成共识的基础上，共同推荐了本肺癌筛查指南。

**结果:**

本指南推荐的肺癌高危人群为：年龄50岁-74岁；吸烟20包/年，或者戒烟5年。参与肺癌LDCT筛查前，需要获得筛查者的知情同意。肺癌筛查需与健康教育结合，向患者宣传吸烟对健康的危害。因此，健康教育应该整合到肺癌筛查全过程，以便帮助患者戒烟。

**结论:**

LDCT筛查能降低肺癌死亡率，本指南推荐中国肺癌高危人群进行LDCT筛查。但是，未来需要进行更多的研究，包括LDCT联合生物标志物用于肺癌筛查的研究，以优化肺癌LDCT筛查方法及技术。

## Abstract (en)

**Background and objective:**

Lung cancer is the leading cause of cancer-related death in China. The results from a randomized controlled trial using annual low-dose computed tomography (LDCT) in specific high-risk groups demonstrated a 20% reduction in lung cancer mortality. The aim of tihs study is to establish the China National lung cancer screening guidelines for clinical practice.

**Methods:**

The China lung cancer early detection and treatment expert group (CLCEDTEG) established the China National Lung Cancer Screening Guideline with multidisciplinary representation including 4 thoracic surgeons, 4 thoracic radiologists, 2 medical oncologists, 2 pulmonologists, 2 pathologist, and 2 epidemiologist. Members have engaged in interdisciplinary collaborations regarding lung cancer screening and clinical care of patients with at risk for lung cancer. The expert group reviewed the literature, including screening trials in the United States and Europe and China, and discussed local best clinical practices in the China. A consensus-based guidelines, China National Lung Cancer Screening Guideline (CNLCSG), was recommended by CLCEDTEG appointed by the National Health and Family Planning Commission, based on results of the National Lung Screening Trial, systematic review of evidence related to LDCT screening, and protocol of lung cancer screening program conducted in rural China.

**Results:**

Annual lung cancer screening with LDCT is recommended for high risk individuals aged 50-74 years who have at least a 20 pack-year smoking history and who currently smoke or have quit within the past five years. Individualized decision making should be conducted before LDCT screening. LDCT screening also represents an opportunity to educate patients as to the health risks of smoking; thus, education should be integrated into the screening process in order to assist smoking cessation.

**Conclusion:**

A lung cancer screening guideline is recommended for the high-risk population in China. Additional research, including LDCT combined with biomarkers, is needed to optimize the approach to low-dose CT screening in the future.

肺癌是全球癌症死亡的首位原因^[1]^。由于人口老龄化和空气污染呈不断加重趋势，且吸烟率居高不下，自21世纪初期，肺癌已成为我国死亡率最高的恶性肿瘤^[2, 3]^。2015年，我国肺癌的发病和死亡例数分别达733, 300人和610, 200人，发病率和死亡率非常接近，其原因主要是由于临床诊断病例多已为晚期，失去了手术机会。肺癌预后极差，我国肺癌的5年生存率仅为16.1%^[4]^。因此，肺癌的早期诊断和早期治疗是提高肺癌生存、降低肺癌死亡率的重要措施^[5]^。

2011年，美国国家肺癌筛查试验（National Lung Screening Trial, NLST）结果表明，在高危人群中，与X线胸片比较，低剂量螺旋CT（Low-dose computed tomography, LDCT）可降低20%的肺癌死亡率，这是首次由随机对照试验（Randomized controlled trail, RCT）显示肺癌可从筛查中获益^[5]^。基于此研究的结果，国际学术组织及国外多家医疗机构已建议在高危人群中开展低剂量螺旋CT筛查，并制定了相应的肺癌筛查指南^[6-14]^。鉴于肺癌严重的疾病负担，我国于2009年在国家医改重大专项“农村癌症早诊早治”项目中将肺癌纳入试点，启动了我国肺癌高危人群筛查工作。目前已涵盖6个省/直辖市的11个肺癌高危人群筛查项目点，每年在20, 000多肺癌高危人群中开展了肺癌LDCT筛查，显著提高了早期肺癌检出率，使我们国家开始有了真正意义上的肺癌筛查早诊早治研究^[15]^。此外，2012年启动的城市早诊早治项目中也包括了肺癌的高危人群筛查^[16]^。因此，探索适合我国国情的肺癌筛查方案也是亟需解决的问题。2015年，我们基于NLST的研究结果及我国肺癌筛查技术方案制定了中国低剂量螺旋CT肺癌筛查指南（2015版）^[17]^。本研究根据我国农村肺癌高危人群筛查项目实践经验，对该指南进行重新修订，以适应国内外肺癌筛查进展。

## 1 背景

近几十年来，肺癌是我国发病和死亡增长最快的恶性肿瘤。根据我国三次死因调查的结果，我国肺癌的年龄标化死亡率从1973年-1975年间的7.30/100, 000增至2004年-2005年间的27.62/100, 000。据估计，2015年我国肺癌新发病例和死亡病例分别占全部肿瘤发病和死亡的17.1%和21.7%^[18]^。总体而言，我国肺癌的发病和死亡率，城市高于农村，男性高于女性，但近年来这种差别正在逐渐缩小。1989年-2008年间，城乡间的肺癌发病率比从2.07降至1.14，而男女发病率比则从2.47降至2.28^[19]^。随着我国肺癌发病率的增高，我国肺癌的住院患者也不断增多。1996年，我国肺癌住院患者为142, 674例，而在2005年则达364, 484例。相应地，肺癌的治疗费用也从1999年的15.47亿元，增至2005年的37.99亿元，年增长率达16.15%^[20]^。因此，肺癌已成为我国危害最为严重的恶性肿瘤，肺癌的防治已成为我国癌症防治的重中之重。

戒烟和控制空气污染是我国目前肺癌一级预防中最重要的两项措施。大量的研究已经表明，戒烟可降低肺癌的发病危险。我国也于2006年签署了《烟草控制框架公约》，并在2012发布了《中国烟草控制规划（2012-2015）》。但近二十年来，我国成年男性吸烟率仍居高不下，而绝大部分吸烟者戒烟的意愿仍较低。因此，通过戒烟来降低肺癌死亡率的效果需要至少几十年才能显现^[21]^。与此类似，我国目前已开始了大气污染的治理，但降低肺癌死亡率的效果仍需时日。

X线胸片和痰细胞学检查曾是最常见的肺癌筛查试验。早期的随机对照研究显示X线胸片可显著提高肺癌的早期检出率、生存率，却并未显示可降低肺癌的死亡率，但这些研究因研究方法学上的缺陷而具有争议^[22]^。美国在前列腺、肺癌、结直肠和卵巢癌筛查试验（Prostate, Lung, Colorectal and Ovarian, PLCO）中对X线胸片筛查的效果再次进行了评估，结果发现X线胸片筛查不能降低肺癌的死亡率^[23]^。

自20世纪90年代起，美国、日本等国家开始对LDCT在肺癌筛查中的作用进行评估，发现LDCT较X线胸片可更为显著地检出早期肺癌，且检出肺癌的生存率极佳^[24]^。基于这些结果，欧美国家开展了多项随机对照试验来评价LDCT是否可降低肺癌死亡率这一金标准。其中，美国NLST是最大的一项，其研究结果表明，经过三轮筛查后，与X线胸片相比，在高危人群中LDCT筛查可降低20%的肺癌死亡率^[5]^。

我国开展的以人群为基础的大规模肺癌筛查是云锡矿工筛查^[25]^。云南省个旧市是亚洲最大的锡工业所在地, 个旧男性肺癌死亡居全国之首。云锡矿工筛查显著提高了早期肺癌检出率和生存率，但因没有对照，同样不能评价X线胸片联合痰细胞学能否降低肺癌死亡率。鉴于我国肺癌的疾病负担日益增加，我国分别于2009年和2012年将肺癌纳入了农村和城市癌症早诊早治项目，并使肺癌高危人群早期肺癌的检出率显著提高。

## 2 材料和方法

本指南由我国国家卫计委肺癌早诊早治专家组及部分从事肺癌临床工作的专家基于2011年肺癌早诊早治技术方案和2015年肺癌筛查指南进行的修订。肺癌早诊早治专家组成员由国家卫计委指定，专业背景涵盖胸外科、肿瘤内科、影像学、病理学、流行病学等学科。

同2015年指南类似，本指南仍基于以下研究结果制订，包括：NLST筛查试验、LDCT筛查系统评价、中国LDCT筛查实践，特别是农村肺癌早诊早治项目^[5, 17, 26, 27]^；国外筛查指南，包括美国胸外科学会（American Association for Thoracic Surgery, AATS）、美国预防服务工作组（U.S.Preventive Services Task Force, UPSTF）、美国肺脏协会（American Lung Association, ALA）、美国胸科医师学会（American College of Chest Physicians, ACCP）、美国癌症学会（American Cancer Society, ACS）、美国国家综合癌症网络（National Comprehensive Cancer Network, NCCN）等机构的肺癌筛查指南^[28]^。

为制定基于证据的肺癌筛查指南，在NLST研究结果发表后，ACS、ACCP、美国临床肿瘤学会（American Society of Clinical Oncology, ASCO）和NCCN对LDCT肺癌筛查进行了一项系统评价。通过文献检索，研究从591篇文献筛选纳入了8项随机对照试验和13项队列研究。基于纳入的研究，对下列4个关键问题进行了评估：LDCT筛查可能的获益；LDCT筛查潜在的危害；可能获益的人群；有效筛查的背景。此系统评价专家组认为此综合性的系统评价可为LDCT筛查指南提供证据基础。

中国农村肺癌早诊早治项目自2010年启动，目前已包括6个省/直辖市的11个项目点，基线筛查和年度筛查的早期肺癌检出率显著高于项目点当地医院的肺癌临床分期。我们主要在此项目点的技术方案修订的基础上对2015年肺癌筛查方案进行修订。

## 3 结果

### 3.1 LDCT扫描参数

本指南建议为：（1）采用螺旋CT容积扫描技术，依据受试者体重，管电压采用100 KVp-140 KVp；管电流 < 60 mAs。总辐射暴露剂量≤5 mSv。（2）扫描范围从肺尖到肋膈角（包括全部肺），患者吸气末一次屏气完成扫描。（3）扫描后原始数据行薄层重建，重建层厚为0.625 mm-1.25 mm。为方便进行计算机辅助检测及容积分析，建议层间有20%-30%重叠。（4）薄层重建算法建议采用软组织密度或肺算法，不建议采用高分辨率骨算法，引起对软件容积分析重复性影响较大。（5）肺结节的检测建议将薄层图像进行三维重建，采用最大密度投影（Maximum intensify projection, MIP）重建，有助于结节的检出及结节形态的观察。推荐应用计算机辅助检测（Computer Aided Design, CAD）软件结合人工阅片，提高结节检出率。

### 3.2 高危人群的选择及筛查间隔

在上述的系统纳入的RCT中，筛查的开始年龄和停止年龄分别介于47岁-60岁和69岁-80岁之间，吸烟量介于15包年-30包年，戒烟最短时间为10年。目前，尚无证据支持开始和停止筛查的具体年龄。在目前唯一证明LDCT筛查有效的NLST试验中，高危人群的纳入标准为：年龄为55岁-74岁，至少有30包/年吸烟史，且如果戒烟，则戒烟时间 < 15年。

在定义筛查的年龄区间时有几个问题需要考虑，包括肺癌的年龄别发病率和预期寿命。在我国，45岁以下的肺癌年龄别发病率相对较低，但随后显著增加，在80岁-84岁时达到高峰。我国男性在50岁-54岁、55岁-59岁、60岁-64岁、65岁-69岁、70岁-74岁、75岁-79岁、80岁-84岁、85岁及以上年龄段的发病率分别为72、139、211、288、408、499、540、465（1/10^5^）^[29]^。2015年，中国人口的预期寿命为76岁^[30]^。此外，75岁-79岁时的预期寿命为9年，如此则只有一半的中国人口预期可存活至84岁。很明显，肺癌的危险随年龄的增加而增大，但由于老年人其他疾病的竞争死亡风险及身体状况的不断下降，很难对年龄为75岁或更大的老人参加LDCT筛查的获益和危害进行权衡。

在8项RCT中，除NELSON试验外，其他研究的LDCT筛查间隔均为1年，而NELSON试验中LDCT筛查时间则为第1年、第2年和第4年。在队列研究中，分别有1项筛查间隔为半年和2年，其他研究则均为每年筛查1次。

基于这些数据，本指南建议参加年度性LDCT筛查的个体为年龄介于50岁-74岁之间的吸烟者，至少有20包/年吸烟史，如已经戒烟则戒烟时间不得超过5年。如果某些高发地区有其他重要的肺癌危险因素也可作为筛选高危人群的条件，如宣威无通风或通风较差室内燃煤年数≥15年；个旧项目点有10年或更长的坑下作业或冶炼史。近5年有癌症病史（非黑色素性皮肤癌、宫颈原位癌、局限性前列腺癌除外）、不能耐受可能的肺癌切除手术或有严重影响生命疾病的个体则不建议进行LDCT筛查。

### 3.3 低剂量螺旋CT筛查中结节的管理

在上述的RCT或队列研究中，关于阳性结节的定义、不同特征结节进一步检查的方法以及是否区分基线筛查和年度筛查方面并不一致。然而，如何确定结节的最佳管理方案来区分肺部良恶性质目前尚无定论。在NLST试验中，直径4 mm及以上的结节需接受进一步检查。本指南中，结节的管理方案采用了中国农村肺癌早诊早治项目的技术方案。

#### 3.3.1 阳性结节的定义

低剂量螺旋CT筛查发现的结节可分为两大类：①肯定良性结节或钙化性结节；②不确定结节或非钙化性结节，此类结节根据结节性质及大小确定随访原则，并根据随访中结节的生长特性确定是否进行临床干预。

基线筛查：若实性结节或部分实性结节直径≥5 mm，或非实性结节直径≥8 mm，或发现气管或/及支气管可疑病变，或低剂量螺旋CT诊断为肺癌的肺部单发、多发结节或肺癌包块，应当进入临床治疗程序则定义为阳性。

年度筛查：发现新的非钙化性结节或气道病变，或发现原有的结节增大或实性成分增加，则定义为阳性。

#### 3.3.2 结节管理和进一步诊断

##### 3.3.2.1 基线筛查检出的结节（图 1）

**1:**
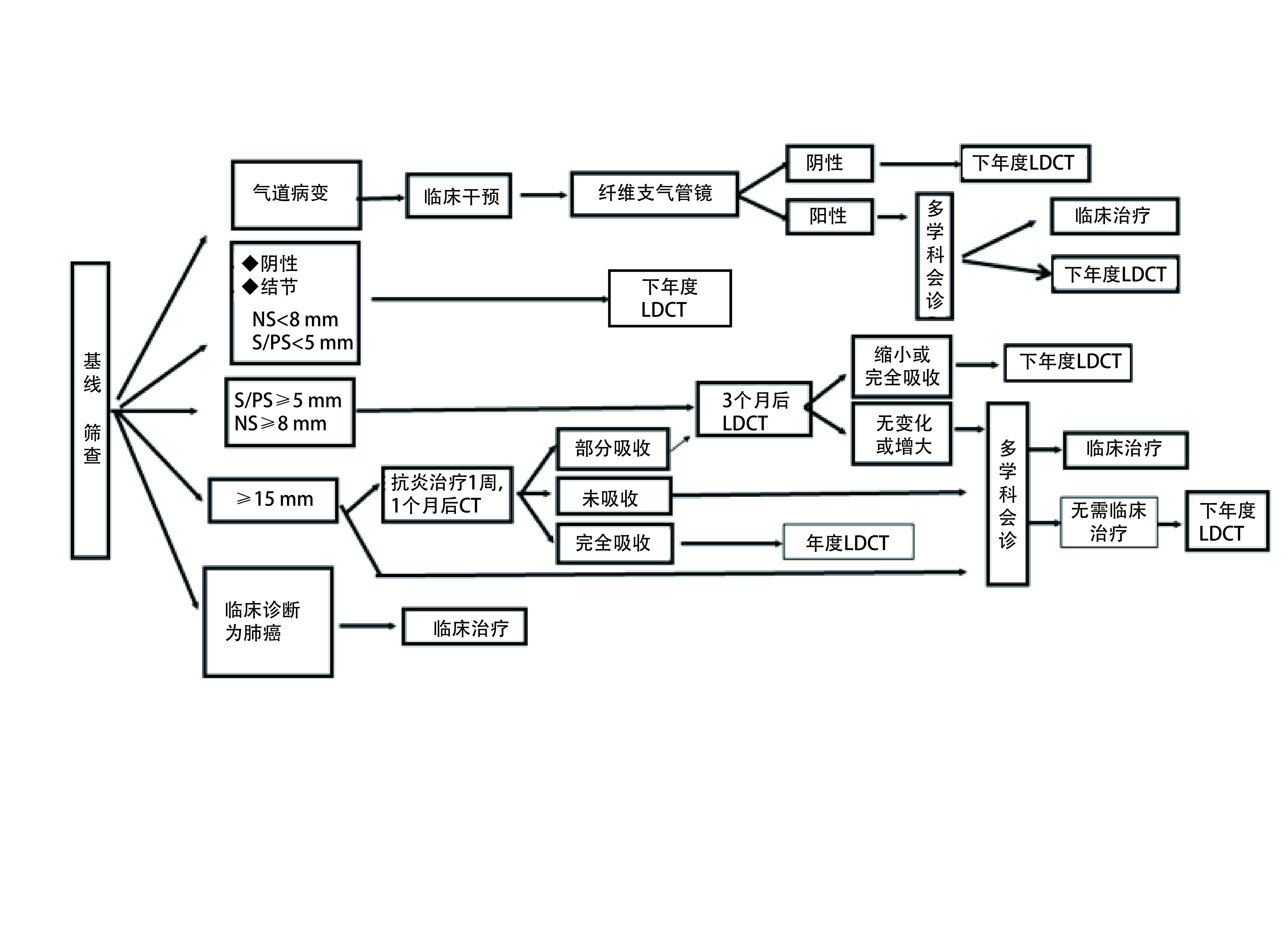
基线筛查流程及结节管理。NS：非实性结节；S：实性结节；PS：部分实性结节。 Baseline screening process and nodule management. ^*^NS: Nonsolid nodule; S: Solid nodules; PS: Partial solid nodules. CT: computed tomography; LDCT: Low-dose computed tomography.

CT检查阴性者， < 5 mm的实性结节或部分实性结节，以及 < 8 mm的非实性结节：12个月后按计划进入下一年度的LDCT复查。5 mm-14 mm的实性结节或部分实性结节，以及8 mm-14 mm非实性结节：筛查后3个月进行LDCT复查。如果结节增大，由多学科高年资医师会诊，决定是否进入临床治疗；如结节无变化，进入下一年度LDCT复查。对于直径≥15 mm结节，有两种方案：①由多学科高年资医师会诊，决定是否进入临床治疗；②抗炎治疗5天-7天，休息1月后复查。如果病灶完全吸收，进入下一年度LDCTCT复查；如果结节无变化，由多学科高年资医师会诊，决定是否进入临床治疗；如果结节部分吸收，3个月后进行LDCT复查，结节增大或无变化者，由多学科高年资医师会诊，决定是否进入临床治疗；结节缩小或完全吸收者，进入下一年度LDCT复查。

低剂量螺旋CT诊断为肺癌的肺部单发、多发结节或肺癌包块，应当进入临床治疗程序。

LDCT筛查如发现气管或/及支气管可疑病变，应进行临床干预，并进行纤维支气管镜检查，并在必要时进一步随诊或者临床治疗。

##### 3.3.2.2 年度筛查结节管理（图 2）

**2:**
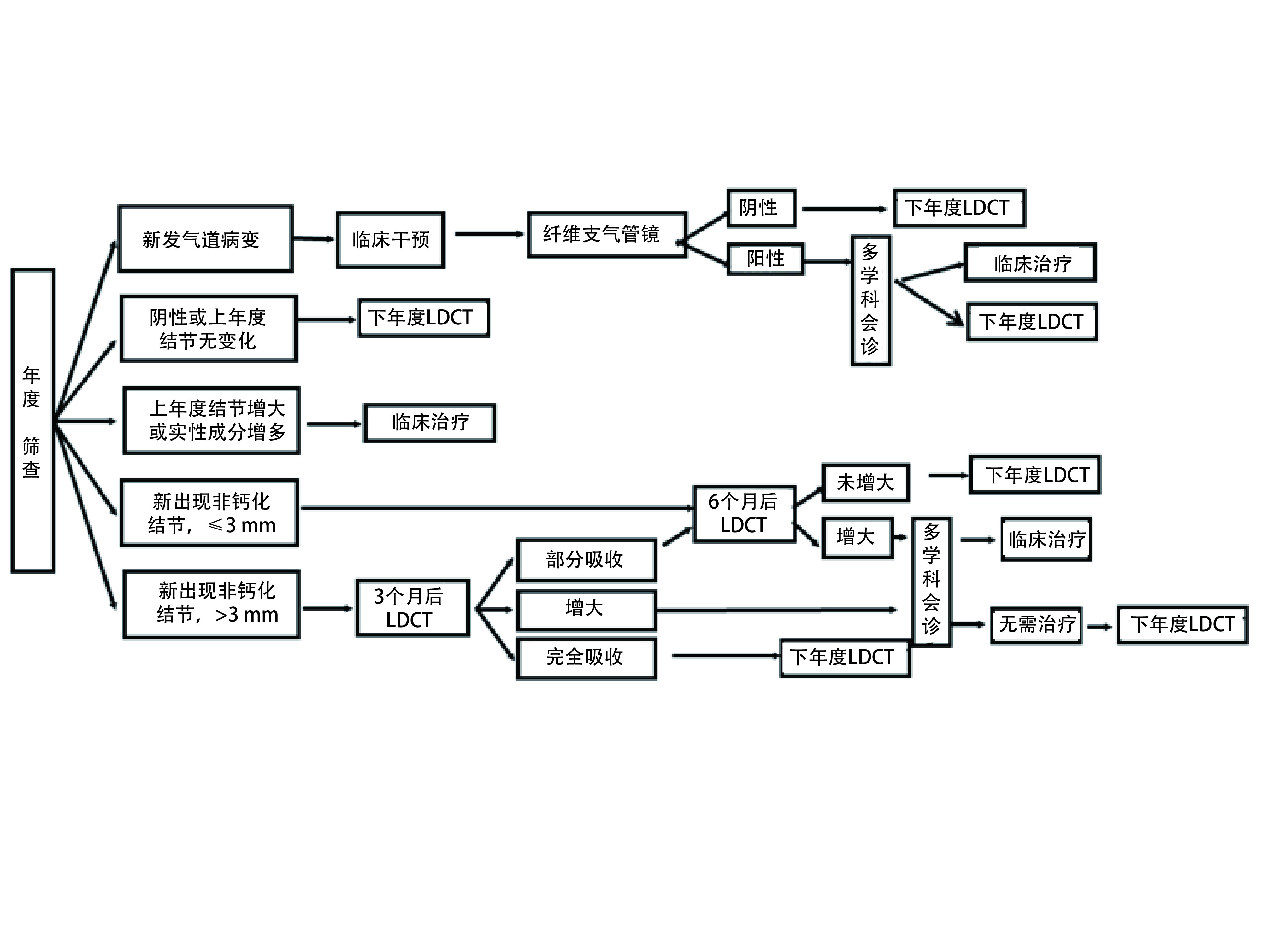
年度筛查流程及结节管理。NS:非实性S:实性结节PS:部分实性结节。 Annual screening process and nodule management. ^*^NS: Nonsolid nodule; S:Solid nodules; PS: Partial solid nodules. CT: computed tomography; LDCT: Low-dose computed tomography.

对于年度LDCT复查发现新的非钙化性结节，若结节直径≤3 mm，6个月后LDCT复查；如果结节增大，由多学科高年资医师会诊，决定是否进入临床治疗程序；如果不需要临床治疗则进入下一年度LDCT复查；如果结节未增大，按计划进入下一年度LDCT复查。

若结节直径 > 3 mm，3个月后LDCT复查若有必要可先进行抗炎治疗，如果随诊中结节增大，由多学科高年资医师会诊，决定是否进入临床治疗；如果结节完全吸收，则进入下一年度LDCT复查；如果结节部分吸收，于6个月后再行LDCT复查；如结节增大，由多学科高年资医师会诊，决定是否进入临床治疗；如果结节无增大，进入下一年度LDCT复查。

年度LDCT复查发现原有的肺部结节明显增大或实性成分明显增多时，应进入临床治疗程序。

年度筛查中发现的气管或/及支气管可疑病变，处理同基线筛查。

#### 3.3.3 临床干预

临床干预包括以下几种情况：

1.低剂量螺旋CT检查发现气道病变者，应该施行纤维支气管镜检查。纤维支气管镜检查阳性，且适合于外科手术治疗者，应当施行外科手术为主的多学科综合治疗。纤维支气管镜检查阴性者，则进入下一年度LDCT复查，或者根据不同情况3个月、6个月后LDCT复查或者纤维支气管镜检查。

2.低剂量螺旋CT诊断为肺癌或高度疑似肺癌者

1）低剂量螺旋CT筛查高度怀疑为肺癌的肺部阳性结节者，应当由高年资的胸外科、肿瘤内科、呼吸科和影像医学科医师集体会诊，决定是否需要进行临床治疗，以及采取什么方法进行治疗。对于适合于外科手术治疗者，一定首选外科治疗。

2）低剂量螺旋CT诊断为肺癌的肺部单发、多发结节或肺癌包块，应当进入临床治疗程序，经临床检查适合外科手术治疗者，施行外科手术为主的多学科综合治疗。

3.低剂量螺旋CT诊断为肺癌或高度怀疑为肺癌的肺部单发、多发结节或肺部包块，由于肿瘤原因、患者心肺功能异常不能耐受外科手术治疗，或者患者本人不愿意接受外科手术治疗者，为明确病变性质进行的经皮肺穿刺活检标本送病理检查及肺癌驱动基因检测。通过经皮肺穿刺活检明确诊断为肺癌者，应当给予化疗为主的多学科综合治疗。

### 3.4 筛查与戒烟结合

多项研究表明，当LDCT筛查发现异常后，肺癌筛查的参与可以给吸烟者戒烟提供机会，建议其戒烟，但这种戒烟教育的效果目前尚无定论^[31, 32]^。但有研究表明，戒烟与年度性LDCT筛查结合可使LDCT筛查的成本效果增加20%-45%^[33]^。ACCP指南中也强调了戒烟的必要性^[28]^。因此，LDCT筛查并非是要替代戒烟，而是要在LDCT筛查中开展戒烟的宣传教育，将二者紧密结合进行。

### 3.5 知情同意与共同决策

虽然LDCT筛查可降低肺癌死亡率，但其仍具有一些潜在的危害，如辐射危险和过高的假阳性结果，进而导致不必要的有创检查。因此，让适合参加筛查的高危个体充分了解LDCT筛查的益处、局限性及潜在的危害非常重要。他们接受LDCT筛查建议前应该与医生或其他医疗专业人员对这些问题进行讨论和共同决策。

## 4 讨论

本指南与2015年版指南比较，本版本主要在基线筛查部分补充了LDCT诊断为肺癌的肺部单发、多发结节或包块，以及明确了在何种情况下进行临床干预。此外，也详细介绍了LDCT扫描参数。这主要是考虑了我国不同级别的医疗机构专业人员的技术水平存在较大差异，有必要根据不同的筛查结果给予特定的随访或干预措施，从而使筛查和干预、治疗路径更为明确。

多数LDCT筛查的研究根据年龄和吸烟量来选择高危人群，然而，越来越多的证据表明，危险预测模型可能有助于更为精准的筛选适合肺癌LDCT筛查的高危个体^[34-36]^。美国一项研究在利用PLCO试验对照的数据建立一种肺癌危险预测模型，并在PLCO筛查组和NLST参与者及美国50岁-80岁的吸烟者中进行了验证，结果发现与USPSTF标准相比，基于模型选择高危人群进行筛查可避免更多的肺癌死亡，并可降低避免肺癌死亡所需的筛查人数^[37]^。NCCN和AATS肺癌筛查指南中也考虑了其他危险因素。将来，需要进一步构建和验证肺癌风险预测模型来提高筛查效果，降低筛查费用。

当建议肺癌高危个体接受LDCT筛查时，也会产生一些潜在的危害^[11]^。其中最突出的是假阳性率过高；不必要且有创的检查，这可能导致患者的损伤；焦虑以及医疗资源的浪费。在我们的筛查建议中，基线筛查阳性结节的最小直径为5 mm，大于NLST试验中的4 mm，这可在保证不影响早期肺癌检出的前提下使结节检出阳性率降低20%。已有报道发现基于患者个体及结节特征来预测筛查检出的肺结节为恶性的统计模型^[38]^。基于年度筛查中结节的体积倍增时间在区分结节的良恶性中也具有重要作用，这种方法已应用于荷兰-比利时肺癌筛查试验（NELSON）^[39]^。将来，我们也将根据这些试验的研究结果来修订本肺癌筛查指南。

肺癌LDCT筛查另一个潜在的危害是辐射，特别是对于肺癌发病危险较低的个体，更应该进行权衡。有研究表明，年龄在50岁以下的人群中，归因于LDCT筛查的死亡率的降低程度并未超过辐射的危险^[40]^。但对于年龄为50岁或更大，且为现在或曾吸烟者（≥20包/年）的肺癌高危个体中，因筛查而产生的死亡率获益大于辐射致癌的危险^[41]^。CT设备和扫描参数设置中技术的进步将会使辐射剂量更低，从而使肺癌LDCT筛查的辐射危险进一步降低。

到目前为止，肺癌LDCT筛查引起的肺癌死亡率的获益仅见于组织性筛查。因此，尚不推荐肺癌的机会性筛查。与NLST试验类似，其他多数RCT和队列研究均在医学研究中心、大型医院中进行，研究团队中包含多学科领域的专家，并制定了明确的纳入标准和结节管理方案。由于不同国家间肺癌的流行情况、医疗保健系统及人群对肺癌筛查的接受程度或依从性并不相同，NLST的研究结果能否在中国推广尚不明确。一些机构已经建议，对于不同地区和国家，可开展示范性项目来评估在本地区开展肺癌LDCT筛查的可行性及相关的不确定性^[28]^。中国已于2010年开展了一项前瞻性、多中心的肺癌筛查示范项目来探讨在中国开展肺癌LDCT筛查的可行性^[15]^。

分子标志物可能有助于鉴别可能因肺癌LDCT筛查获益的肺癌高危人群，从而降低筛查费用，减少筛查相关的危害。此外，分子标志物和肺癌LDCT筛查的联合应用可能降低过高的假阳性结果^[42]^。NLST试验已经建立了包括血、痰和尿液样本的生物样品库，以便发现和验证确定肺癌高危个体、区分结节良恶性，以及预测肿瘤生物学行为的标志物^[43]^。但目前研究中报道的大量肺癌早期标志物的诊断性能尚需基于大样本量人群的前瞻性研究验证。

综上所述，目前肺癌已成为我国癌症死亡的首位原因，且预后极差。肺癌LDCT筛查获益已得到随机对照试验研究的证实。为使获益最大化并减少LDCT所致伤害，肺癌LDCT筛查应以组织性筛查的方式开展，并且肺癌高危个体在参加LDCT筛查之前，应与医生或其他医疗专业人员对这些问题进行讨论和共同决策。如何筛选高危人群，如何寻找LDCT筛查出的肺部结节的最佳管理策略，是未来提高肺癌LDCT筛查获益，减少潜在危害的关键环节，我国应在这些方面积极开展相应的前瞻性研究。
